# Biochemical Studies of Mitochondrial Malate: Quinone Oxidoreductase from *Toxoplasma gondii*

**DOI:** 10.3390/ijms22157830

**Published:** 2021-07-22

**Authors:** Rajib Acharjee, Keith K. Talaam, Endah D. Hartuti, Yuichi Matsuo, Takaya Sakura, Bundutidi M. Gloria, Shinya Hidano, Yasutoshi Kido, Mihoko Mori, Kazuro Shiomi, Masakazu Sekijima, Tomoyoshi Nozaki, Kousuke Umeda, Yoshifumi Nishikawa, Shinjiro Hamano, Kiyoshi Kita, Daniel K. Inaoka

**Affiliations:** 1 Program for Nurturing Global Leaders in Tropical and Emerging Communicable Disease, Graduate School of Biomedical Science, Nagasaki University, Nagasaki 852-8523, Japan; rajibacharjee@cu.ac.bd (R.A.); keithtalamk@gmail.com (K.K.T.); endah.dwi08@yahoo.co.id (E.D.H.); gloriadea03@gmail.com (B.M.G.); shinjiro@nagasaki-u.ac.jp (S.H.); 2 Department of Parasitology, Institute of Tropical Medicine (NEKKEN), Nagasaki University, Nagasaki 852-8523, Japan; 3 Department of Zoology, University of Chittagong, Chittagong 4331, Bangladesh; 4 Laboratory for Biotechnology, Agency for the Assessment and Application of Technology, South Tangerang 15314, Indonesia; 5 School of Tropical Medicine and Global Health, Nagasaki University, Nagasaki 852-8523, Japan; ymatsuo@kumamoto-u.ac.jp (Y.M.); takaya.sakura@nagasaki-u.ac.jp (T.S.); kitak@nagasaki-u.ac.jp (K.K.); 6 Graduate School of Life Science, Kumamoto University, Kumamoto 860-0862, Japan; 7 Department of Molecular Infection Dynamics, Institute of Tropical Medicine (NEKKEN), Nagasaki University, Nagasaki 852-8523, Japan; 8 Department of Pediatric, Kinshasa University Hospital, University of Kinshasa, Kinshasa P.O. Box 123, Congo; 9 Department of Immune Regulation, The Research Center for Hepatitis and Immunology, National Center for Global Health and Medicine, Chiba 272-8516, Japan; lb-21hidano@hospk.ncgm.go.jp; 10 Department of Parasitology and Research Center for Infectious Disease Sciences, Graduate School of Medicine, Osaka City University, Osaka 545-8585, Japan; kido.yasutoshi@med.osaka-cu.ac.jp; 11 Biological Resource Center, NITE, Kisarazu, Chiba 292-0818, Japan; mori-mihoko@nite.go.jp; 12 Graduate School of Infection Control Sciences, Kitasato University, Tokyo 108-0072, Japan; shiomi@lisci.kitasato-u.ac.jp; 13 Department of Advanced Computational Drug Discovery Unit, Tokyo Institute of Technology, Yokohama 226-8501, Japan; sekijima@c.titech.ac.jp; 14 Department of Biomedical Chemistry, Graduate School of Medicine, The University of Tokyo, Tokyo 113-0033, Japan; nozaki@m.u-tokyo.ac.jp; 15 Pathology Division, Aquaculture Research Department, Fisheries Technology Institute, Japan Fisheries Research and Education Agency, Minamiise 516-0193, Japan; umeda_kousuke97@fra.go.jp; 16 Research Unit for Host Defense, National Research Center for Protozoan Diseases, Obihiro University of Agriculture and Veterinary Medicine, Obihiro 080-8555, Japan; nisikawa@obihiro.ac.jp; 17 The Joint Usage/Research Center on Tropical Disease, Institute of Tropical Medicine (NEKKEN), Nagasaki University, Nagasaki 852-8523, Japan; 18 Department of Host-Defense Biochemistry, Institute of Tropical Medicine (NEKKEN), Nagasaki University, Nagasaki 852-8523, Japan

**Keywords:** toxoplasmosis, electron transport chain, mitochondria, membrane protein, enzyme inhibition, ferulenol

## Abstract

*Toxoplasma gondii* is a protozoan parasite that causes toxoplasmosis and infects almost one-third of the global human population. A lack of effective drugs and vaccines and the emergence of drug resistant parasites highlight the need for the development of new drugs. The mitochondrial electron transport chain (ETC) is an essential pathway for energy metabolism and the survival of *T. gondii*. In apicomplexan parasites, malate:quinone oxidoreductase (MQO) is a monotopic membrane protein belonging to the ETC and a key member of the tricarboxylic acid cycle, and has recently been suggested to play a role in the fumarate cycle, which is required for the cytosolic purine salvage pathway. In *T. gondii*, a putative MQO (TgMQO) is expressed in tachyzoite and bradyzoite stages and is considered to be a potential drug target since its orthologue is not conserved in mammalian hosts. As a first step towards the evaluation of TgMQO as a drug target candidate, in this study, we developed a new expression system for TgMQO in FN102(DE3)TAO, a strain deficient in respiratory cytochromes and dependent on an alternative oxidase. This system allowed, for the first time, the expression and purification of a mitochondrial MQO family enzyme, which was used for steady-state kinetics and substrate specificity analyses. Ferulenol, the only known MQO inhibitor, also inhibited TgMQO at IC_50_ of 0.822 μM, and displayed different inhibition kinetics compared to *Plasmodium falciparum* MQO. Furthermore, our analysis indicated the presence of a third binding site for ferulenol that is distinct from the ubiquinone and malate sites.

## 1. Introduction

Toxoplasmosis is a zoonosis caused by *Toxoplasma gondii*, an obligate intracellular apicomplexan protozoan parasite that can infect almost all nucleated cells [1,2,3,4]. Recent estimations are that 30–50% of the global population is seropositive for *T. gondii* [5]. It has long been considered a mild pathogen compared to other deadly apicomplexan parasites such as *Plasmodium falciparum* [6], the pathogen of malaria, though in many aspects *T. gondii* exhibits metabolic traits similar to those of *Plasmodium* species, particularly with regard to the hepatic stage [7]. The capacity of *T. gondii* to adapt to different environmental parameters, such as pH [8,9], temperature, oxidative and chemical stresses [8,10], during both sexual and asexual stages in a wide range of mammalian hosts, including humans, ranks *T. gondii* as one of the most successful parasites [11].

A correlation between the geographical variations of *T. gondii* genotypes and disease manifestation in humans has been established from population genetics and epidemiological studies [11,12,13]. Among three known and well-characterized lineages of *T. gondii* (types I, II, and III), types I and II are predominantly distributed in North America and type II in Europe [14]. The seroprevalence is high [15], perhaps due to ease of transmission of *T. gondii*, reaching 90% in some European and South American countries, which is skewed down to 22.5% in the United States [5]. Typically, the disease is asymptomatic in immunocompetent hosts, but provokes severe illness in immunocompromised patients such as those with acquired immunodeficiency syndrome, pregnant women, or congenitally infected individuals [16,17]; without treatment, it can lead to death [18]. To date, toxoplasmosis chemotherapy options are limited, and all drugs currently used have severe side effects, are solely active against tachyzoites, and not able to clear cystic chronic infection [19,20]. Furthermore, Toxovax, the only vaccine for animal toxoplasmosis, is not suitable for humans because of iatrogenic infection risks and interconversion of parasite stages [21,22]. Therefore, it is needed to develop new drugs for both tachyzoites and bradyzoites in cysts with novel mechanisms of action with fewer side effects [19]. Undoubtedly, metabolic pathways or molecular targets from the parasites that are absent or different from the corresponding host pathways are attractive drug targets [19,23].

Although glucose and glutamine are the major carbon sources, the metabolic plasticity of *T. gondii* supports its survival in a wide host range [24,25]. However, to support the optimal growth of *T. gondii* tachyzoites and effective pathogenesis in mammalian hosts, glucose is required and cannot be complemented by other carbon sources, such as glutamine, succinate, pyruvate, glycerol, or propionate [26]. Previous studies have revealed the presence of a functional tricarboxylic acid (TCA) cycle in tachyzoites [24,27]. Moreover, the comparative expression analysis of TCA cycle enzymes has shown similar mRNA levels between tachyzoites and early bradyzoites [27]. Collectively, these findings suggest that, to meet its energy demands, this parasite acquires adenosine triphosphate (ATP) not only by glycolysis but also by oxidative phosphorylation.

The mitochondrial electron transport chain (ETC) has been proven to be a potential target for drug development against parasites [28,29,30,31]. For example, in the blood stream form of *Trypanosoma brucei*, the protozoan parasite that causes sleeping sickness, its ETC is composed mainly of glycerol-3-phosphate dehydrogenase (G3PDH) and a cyanide-insensitive trypanosome alternative oxidase (TAO). TAO is a terminal oxidase not conserved in mammalian hosts and a validated drug target for the development of species-selective drugs [32,33,34,35]. Similar to all eukaryotes, the mitochondria of the apicomplexan parasite are involved in several essential cellular metabolic pathways and serve as important drug target organelles [23,36]. Although apicomplexan parasites possess the smallest mitochondrial genomes amongst all analyzed eukaryotes [37], their mitochondria contain almost 400 putative mitochondrial matrix proteins and several inner membrane proteins [36], such as the *bc*_1_ complex (complex III) and dihydroorotate dehydrogenase (DHODH), which are important for growth and survival [38,39,40].

Similar to other apicomplexans, five types of ETC dehydrogenase are coded in the *T. gondii* genome: type II nicotinamide adenine dinucleotide hydrogen (NADH) dehydrogenase (TgNDH2-I and TgNDH2-II) [41], succinate dehydrogenase (TgSDH or complex II) [27,42], and malate:quinone oxidoreductase (TgMQO) [43] are involved in the TCA cycle; TgG3PDH [41,44] is involved in lipid metabolism and redox homeostasis [45,46]; DHODH (TgDHODH) [47] catalyzes the fourth step in pyrimidine de novo biosynthesis. All these ETC dehydrogenases transfer electrons from their individual substrates to the ubiquinone-pool (UQ-pool). The electrons from the UQ-pool are sequentially transferred to cytochrome *c* and molecular oxygen (O_2_) through reactions catalyzed by complex III and cytochrome *c* oxidase (complex IV), respectively. During electron transport, protons are translocated from the mitochondrial matrix to the intermembrane space by complexes III and IV to maintain an electrochemical gradient that is used by ATP synthase (complex V) for ATP synthesis [40]. Inhibitors of complex III, such as atovaquone [48] and endochin-like quinolone [48], are known to inhibit the proliferation of several lifecycle stages of *T. gondii* [49].

In contrast to *P. falciparum*, *T. gondii* seems to have the ability to obtain pyrimidine via the salvage pathway [50] in addition to the de novo pathway. *T. gondii* mutants auxotrophic for uracil, a salvage pathway precursor, can be obtained by knockout of carbamoyl phosphate synthetase II (CPSII, the first step of the de novo pathway); however, they are avirulent in animal models [51]. Attempts to generate DHODH (the fourth step of the de novo pathway) null mutants have failed even in the presence of uracil, although uracil auxotrophic mutants can be obtained by gene replacement of endogenous DHODH with a catalytically deficient version [47]. Collectively, these findings suggest that, in *T. gondii*, CPSII and DHODH might have additional roles, other than de novo pyrimidine biosynthesis, such as in parasite virulence [51] and ETC integrity [47], respectively.

MQO is widely conserved in bacteria and in some unicellular eukaryotes; however, it is absent in mammalian hosts. MQO catalyzes the irreversible conversion of malate to oxaloacetate [52] and is a key member of the TCA cycle. MQO is catalytically equivalent to the reversible mitochondrial malate dehydrogenase (mMDH) from mammals, but it differs in localization and in its electron acceptor. MQO is localized in the mitochondrial inner membrane and is linked to the ETC by using ubiquinone as the electron acceptor. In contrast, mMDH is a matrix protein that uses nicotinamide adenine dinucleotide (NAD^+^) as the electron acceptor. In apicomplexan parasites, the role of MQO is not completely understood, and in addition to the TCA and ETC [43], it has been suggested that MQO provides cytosolic oxaloacetate to feed the purine salvage and pyrimidine de novo pathways in the recently identified fumarate cycle [53,54]. Because MQO is conserved in some organisms with incomplete TCA cycles, such as *Cryptosporidium parvum*, it has been speculated that, in addition to its functional role, MQO can potentially have a structural role [55,56].

Nonetheless, a putative MQO (DQ457183) is conserved in the *T. gondii* genome, which shows high similarity to MQOs from ε-proteobacteria [23], and thus it belongs to MQO family group 2 [57]. Interestingly, in addition to MQO, a putative mMDH (EF683092) can be found in its genome. In general, malate oxidation by MDHs is thermodynamically unfavorable, and the reduction of oxaloacetate by NADH is preferred [58]. In the presence of MQO and MDH, it has been proposed that they act in antagonistic directions [59]. However, forced oxidation of malate to oxaloacetate by MDH can be anticipated at unphysiologically high malate concentrations [60].

Ferulenol, the first inhibitor of MQO identified, inhibits the growth of *P. falciparum* and shows synergism in combination with atovaquone [23]. Moreover, the growth of mqo-disrupted *P. berghei* is impaired, and the parasite is unable to cause cerebral malaria in a mouse model [61], indicating that MQO plays essential roles in optimum growth and virulence. In addition, because of the trifunctional role of MQO in the ETC, TCA, and fumarate cycle, it is considered as a potent pharmacological target against *P. falciparum* [23].

We have previously reported the development of a recombinant *P. falciparum* MQO (PfMQO) expression system in a bacterial membrane [BL21(DE3)], its biochemical characterization, and the identification of inhibitors [23,62]. In this report, we describe a new expression system for recombinant TgMQO in FN102(DE3)TAO (Figure S1), and for the first time, the steady-state kinetics and substrate specificity towards electron acceptors of a purified mitochondrial MQO family enzyme.

## 2. Results

### 2.1. Functional Expression and Purification of Recombinant TgMQO

TgMQO was successfully overexpressed in the membrane fractions of BL21(DE3) and FN102(DE3)TAO. Moreover, the specific activity of TgMQO was higher in FN102(DE3)TAO (6.4 ± 0.6 μmol/min/mg) than in BL21(DE3) (2.84 ± 0.16 μmol/min/mg) membrane fractions. In addition, the specific activity of TgMQO increased to 7.5 ± 0.2 μmol/min/mg in the presence of ascofuranone (AF), a specific TAO inhibitor (Figure S1) [63,64]. The specific activity of TgMQO was high in the membrane fraction prepared using HEPES buffer, but a fast decrease was observed after solubilization, making this buffer unfit for purification. Compared to the HEPES buffer, we found that the specific activity of TgMQO prepared with MOPS buffer was lower in the membrane fraction but higher after purification and stable for several months (Table S1). Under optimized conditions, 3.7 mg of purified TgMQO was obtained from a 3-L culture of FN102(DE3)TAO. The purification yield was 7%, and the specific activity was 22 μmol/min/mg protein (measured at 37°C), corresponding to a 10.5-fold increase compared with the lysate (Table S1). The specific activity of MQO in the membrane fractions was about 145-fold higher in FN102(DE3)TAO/TgMQO (2.9 μmol/min/mg) than the endogenous MQO activity (0.02 μmol/min/mg) (Table S1). Judging from sodium dodecyl sulphate–polyacrylamide gel electrophoresis (SDS-PAGE) (Figure S2a), the purity of TgMQO was over 90%, and according to the migration length, it had a molecular weight of 61 kDa (Figure S2a,b).

**Figure 1:**
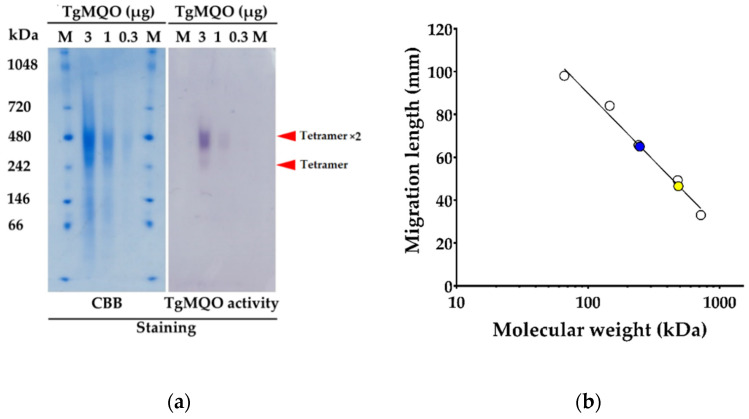
High resolution clear native electrophoresis (hrCNE) of TgMQO. (**a**) Coomassie brilliant blue (CBB) and activity staining of TgMQO. The red arrows show visible bands by both GelCode Blue and activity stainings, corresponding to tetramer and dimer of tetramer of TgMQO, respectively. M = NativeMark^TM^ protein standard, 5 μL. (**b**) Logarithmic plot (migration length vs. molecular weight) from hrCNE. The blue and yellow dots represent the molecular weights of tetramer (247 kDa) and dimer of tetramer (486 kDa) of TgMQO, respectively.

Under the high resolution clear native electrophoresis (hrCNE), two bands of TgMQO were clearly visible by GelCode Blue and activity stainings (Figure 1a). The lower (247 kDa) and higher (486 kDa) molecular weight bands (Figure 1a,b) coincide with a tetramer and a dimer of tetramer, respectively.

### 2.2. Biochemical Characterization of Purified TgMQO

**Figure 2:**
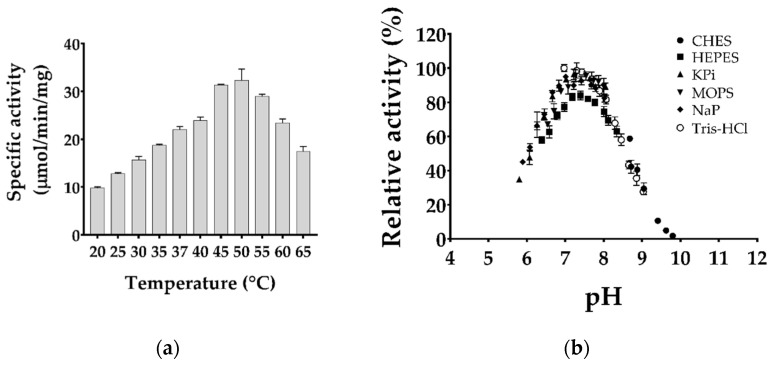
Optimization of purified TgMQO activity conditions. (**a**) TgMQO activity was assayed spectrophotometrically at varying temperatures (°C) in triplicate. Optimum temperature was found to be higher (50 °C) than normal physiological temperature (37 °C). (**b**) pH optimization was performed in a plate-reader under different pH conditions at 37 °C and 600 nm in quadruplicate. Insignificant differences in TgMQO activity were observed for pH ranging from 7.0 to 8.0.

Purified TgMQO was used to determine the dose response, optimum temperature, and pH for MQO activity. Dose response assays showed a linear response of TgMQO activity from 0.025 µg/mL to 2.5 µg/mL in the assay mixture with an R^2^ value of 0.9997 and minimal change in the specific activity (Figure S3). The optimum temperature for TgMQO enzyme activity was found to be 50 °C (Figure 2a), then started to decrease. However, at ambient temperatures of 25 and 30 °C, and a physiological temperature of 37 °C, the enzyme retained around 40%, 50%, and 70% of its maximum activity at 50 °C, respectively. The optimum pH for TgMQO was determined under physiological temperature (37 °C) and, as shown in Figure 2b, it displayed a bell-shaped response and an apparent optimum pH with Tris-HCl 7.0; however, no significant difference was observed within a pH range of 7.0 to 8.0. The concentration of TgMQO was fixed at 0.0375 µg/mL, and for better comparison with PfMQO, HEPES pH 7.0 and 37 °C were further used to determine the kinetic parameters.

**Figure 3:**
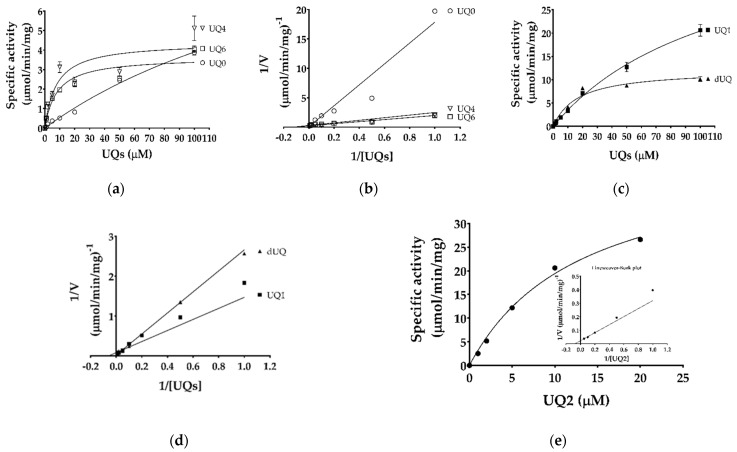
Kinetics of purified TgMQO with different ubiquinones. Michaelis-Menten and Lineweaver-Burk lines for UQ0, UQ4, and UQ6 (**a**,**b**); UQ1 and dUQ (**c**,**d**); and UQ2 (**e**). In each case, the reaction was initiated with 10 mM malate. The kinetic constants (*K*_m_ and *V*_max_) calculated for the ubiquinones are summarized in Table 1. Amongst the ubiquinones, UQ2 showed substrate inhibition over 20 μM.

**Figure 4:**
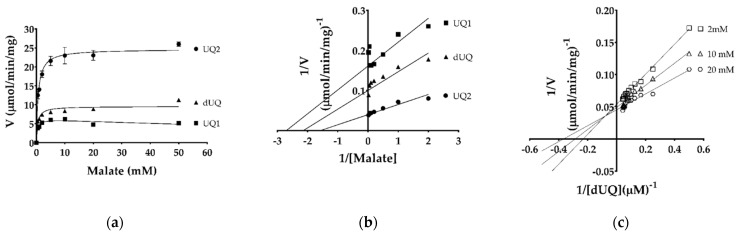
Determination of kinetic parameters of purified TgMQO by varying malate concentrations. (**a**) Michaelis-Menten curves and (**b**) Lineweaver-Burk plots of malate for ubiquinones (UQ1, UQ2, and dUQ). (**c**) The reaction mechanism catalyzed by purified TgMQO was analyzed by double reciprocal plots of TgMQO activity at varying dUQ concentrations under fixed malate concentrations of 2, 10, and 20 mM. The resulting lines intersecting above the X-axis in the second quadrant indicate a sequential mechanism of the reaction.

The kinetic parameters for different ubiquinones and malate were determined by monitoring ubiquinone reduction at 278 nm. The affinity (*K*_m_) values for different ubiquinones were 225, 116, 13.2, 7.7, 8.0, and 17 µM, whereas *V*_max_ were 12.7, 44.2, 45.1, 4.39, 3.64, and 12.1 µmol/min/mg for UQ0, UQ1, UQ2, UQ4, UQ6, and decyl-UQ (dUQ), respectively (Table 1, Figure 3a–e). This indicates that TgMQO has a higher affinity to quinones with longer side chains, such as UQ2, UQ4, UQ6, and dUQ, compared to short ones. Interestingly, substrate inhibition was observed for UQ2 even at 20 µM (Figure S4), but not for UQ4 and UQ6, possibly because of their low solubility in our assay conditions (Figure 3a). The *K*_m_ for malate showed little variation between UQ1, UQ2, and dUQ (370, 637, and 466 µM, respectively) (Table 1, Figure 4a,b).

The reaction mechanism of purified TgMQO was investigated by varying the concentration of dUQ under fixed concentrations of malate at 2, 10, and 20 mM. Our analysis showed that the lines derived from double reciprocal plots are not parallel but intersect in the second quadrant, supporting a sequential (random or ordered) reaction mechanism for TgMQO (Figure 4c).

### 2.3. IC_50_ and Inhibition Mechanism of Ferulenol with TgMQO

**Figure 5:**
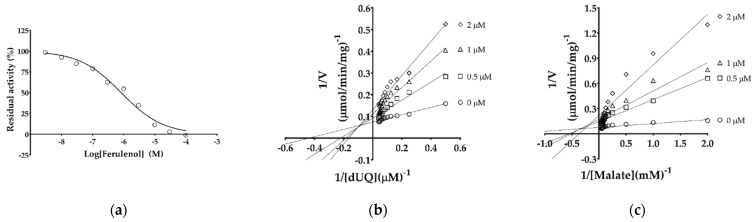
Inhibition of purified TgMQO activity by ferulenol. (**a**) TgMQO activity was assayed in the presence of serially diluted ferulenol concentrations at 278 nm, and activity was recorded as residual activity (%). The inhibition mechanism of ferulenol was also determined (**b**) vs. dUQ and (**c**) vs. malate. In both cases, the double reciprocal lines intersected in the second quadrant, indicating a mixed type inhibition of TgMQO activity.

Ferulenol was previously identified as the first inhibitor of MQO family enzymes, displaying an IC_50_ of 57 nM against PfMQO [23]. To test the effect of ferulenol on TgMQO, the IC_50_ was determined as 0.822 ± 0.151 µM (Figure 5a).

Next, the inhibition mechanism of ferulenol was analyzed, and as shown in Figure 5b, double reciprocal plots obtained by varying the concentrations of dUQ yielded straight lines, which intersected in the second quadrant, suggesting a mixed type of inhibition. The same experiment performed by varying malate concentrations yielded similar results as described above, indicating a mixed type inhibition also for malate (Figure 5c).

## 3. Discussion

We previously reported an expression system for recombinant PfMQO overexpressed in the membrane fraction of BL21(DE3) with high specific activity [23]. Since the purification of PfMQO was unsuccessful, the biochemical characterization was performed using membrane fractions in the presence of potassium cyanide (KCN). *Escherichia coli* contains several terminal quinol oxidases, such as cytochrome *bo*_3_, *bd*-I, and *bd*-II. Because *bd*-I and *bd*-II are KCN-insensitive oxidases [65,66,67], the activity of any enzyme using a quinone/quinol-dependent assay system would be not accurate because of quinol consumption by those cytochromes (Figure S1). To avoid this problem, TgMQO was expressed in FN102(DE3)TAO, because this strain lacks glutamyl-tRNA reductase (hemA), the first step of the heme biosynthesis pathway [68]. Because all terminal quinol oxidases from *E. coli* are heme dependent, the FN102(DE3) strain is unable to re-oxidize quinols and grow unless TAO, a non-heme diiron quinol oxidase, is expressed. Unlike heme-dependent terminal quinol oxidases from *E. coli*, TAO is specifically and potently inhibited by AF. In this study, TAO was expressed in pACYC because it is compatible with the pET system. Interestingly, the specific activity of TgMQO expressed in FN102(DE3)TAO was higher than that from the BL21(DE3) system (Figure S1). As expected, the TgMQO specific activity increased from 6.4 to 7.5 µmol/min/mg when assayed in the absence and presence of 50 nM AF, respectively (Figure S1). This clearly demonstrates that the quinone/quinol-dependent activity assayed in the conventional *E. coli* expression system was underestimated, and FN102(DE3)TAO is an excellent alternative expression system to study membrane-bound ETC dehydrogenases. Using this new expression system, for the first time, we purified the mitochondrial type MQO in milligram amounts with high specific activity ranging from 15-26 µmol/min/mg depending on the lot (Table S1). Several bacterial types of MQO have been purified, such as from *Geobacillus thermodenitrificans* DSM 465 (0.12 µmol/min/mg) [69], *Acetobacter* sp. SKU 14 (15 µmol/min/mg) [70], *Corynebacterium glutamicum* (~10 µmol/min/mg) [58], *Pseudomonas taetrolens* (9.3 μmol/min/mg) [71], and *Bacillus* sp. PS3 (24.6 μmol/min/mg) [72]. However, no crystal structures have been reported.

Purified bacterial MQOs display variations in their oligomerization state, such as homo-dimers (*Bacillus* sp. PS3 and *Acetobacter* sp. SKU 14) and a decamer (*G. thermodenitrificans* DSM 465) [69,70,72]. Here, we provide the first evidence of the oligomeric state of mitochondrial type MQO in solution by hrCNE followed by MQO activity and CBB stainings (Figure 1a,b). As shown in Figure 1a, two bands corresponding to a tetramer (247 kDa) and a dimer of tetramer (486 kDa) of TgMQO were observed, the latter being more active than the tetrameric form.

In the present study, we noticed that 2,6-dichlorophenolindophenol (DCIP)-linked activity was higher than the direct detection of dUQ consumption, which was not observed for UQ2 (Figure S5). Moreover, the reduction of DCIP in the absence of quinones (4.2 µmol/min/mg) indicates that DCIP might act directly as an electron acceptor by binding at malate and/or ubiquinone binding sites, increasing the specific activity in the presence of dUQ. The lower rate of DCIP reduction in presence of UQ2 compared to dUQ can be explained by competition for electrons between UQ2 and DCIP, which is not observed between dUQ and DCIP (Figure S5). Collectively, our data support the binding of DCIP at the ubiquinone rather than malate binding site.

Similar to PfMQO, TgMQO showed an optimum pH range within pH 7.0 to 8.0, with small variations [23]. It has been demonstrated that the optimum temperature of PfMQO is 37 °C. Surprisingly, our analysis showed that TgMQO displayed an optimum temperature for its activity at 50 °C (Figure 2a). This may suggest that under physiological conditions, *T. gondii* mitochondria are maintained at 50 °C, as recently suggested for respiratory complex enzymes (complex III, II-III, IV, and V) in mitochondria of human cells [73,74], or is the result of adaptation to mitochondrial recruitment after invasion [75,76,77,78].

The kinetic parameters of active and purified TgMQO were determined and are summarized in Table 1. TgMQO showed a similar affinity to malate using three different quinones as electron acceptors (*K*_m_ 370, 637, and 466 μM for UQ1, UQ2, and dUQ, respectively). Those values were 10–15 times lower than the reported *K*_m_ of PfMQO (5990 ± 340 μM using dUQ) [23]. For the electron acceptor, TgMQO showed considerably high affinity (*K*_m_ ranging from 7 to 17 μM) to quinones with long side chains (UQ2, UQ4, UQ6, and dUQ), while for short chain quinones (UQ0 and UQ1), it displayed low affinity (*K*_m_ 116 and 225 μM). Interestingly, unlike malate, the affinity of dUQ (*K*_m_ = 17 μM) to TgMQO was approximately three times lower than the reported affinity to PfMQO (6.2 ± 0.65 μM) [23]. According to our results, TgMQO employs a sequential reaction mechanism that is similar to that found for PfMQO [23], suggesting that this kind of reaction mechanism is conserved among mitochondrial-type MQOs.

Ferulenol is a sesquiterpene prenylated coumarin isolated from *Ferula communis* [79] that is reported to have antibacterial [80], anti-coagulant [81,82,83], and anti-cancer [84] activities. Several targets of ferulenol have been identified so far, such as vitamin K epoxide reductase of rat [85] and bacteria [86], complex II [79,87], PfMQO [23], *Eimeria tenella* DHODH [88], TAO, trypanosomal glycerol kinase [34,89,90], and human DHODH [88]. In this study, we showed that ferulenol is also an inhibitor of TgMQO; however, its IC_50_ was 14 times higher (0.822 ± 0.151 μM) than that of PfMQO (0.057 μM) (Figure 5a) [23]. Interestingly, with TgMQO, ferulenol showed a mixed type of inhibition for malate (α = 646.8) and dUQ (α = 27.5) (Figure 5b,c). This indicates that ferulenol may bind to TgMQO as well as TgMQO-dUQ or TgMQO-malate complexes [91], showing higher affinity to free TgMQO than to the TgMQO-substrate complex. This finding reinforces the notion that dUQ, malate, and ferulenol have different binding sites in TgMQO as described for PfMQO [23], but without the formation of a dead-end complex (TgMQO-dUQ-ferulenol). These patterns of inhibition by ferulenol are more consistent with a sequential ordered mechanism in which quinone binds first and then malate, similar to the mechanism described for PfMQO [23]. Nevertheless, the mixed type of inhibition of ferulenol for both substrates is a desirable feature for drug development, because it can bind to TgMQO and TgMQO-substrate complexes, hence inhibiting the enzyme function regardless of substrate concentrations in the parasite.

Finally, we identified ferulenol as the first mixed type of inhibitor of MQO, though the IC_50_ was in the lower µM range. As MQOs are conserved among different apicomplexan parasites and not present in their mammalian hosts, this might help to design drug candidates with novel mechanisms of action and a broad spectrum of activity with fewer side effects.

## 4. Materials and Methods

### 4.1. Bacterial Strains, Plasmids, and Reagents for Recombinant TgMQO Expression

The TgMQO gene (DQ457183), lacking the mitochondrial targeting signal (MTS; Δ1-37 residues), was codon optimized for expression in *E. coli* and cloned into pET151-D-TOPO (Invitrogen, Carlsbad, CA, USA) to generate pET151/TgMQO, according to the manufacturer’s protocol.

The gene coding for TAO was amplified from pET101/NHis6SUMO-ΔMTS-TAO [92] and cloned into pACYC-Duet (pACYC-TAO). After the sequence was confirmed, this plasmid was used to transform FN102(DE3), a heme-deficient strain unable to re-oxidize ubiquinol unless 5-aminolevulinic acid (ALA) is provided or TAO is expressed [68]. The resultant strain, FN102(DE3)TAO, was used as the expression host and transformed with pET151/TgMQO. The final strain, named FN102(DE3)TAO/TgMQO, was selected on Luria-Bertani (LB) agar plates supplemented with 100 μg/mL carbenicillin (Wako, Kanagawa, Japan), 50 μg/mL kanamycin (Wako), 50 μg/mL chloramphenicol (TCI, Zwijndrecht, Belgium), and 50 μg/mL ALA.

### 4.2. Overexpression of Active TgMQO in the Bacterial Membrane

A frozen stock of FN102(DE3)TAO/TgMQO in 20% (*v/v*) glycerol (Wako) was used to streak LB agar plates containing antibiotics and ALA, then kept at 37 °C overnight. Colonies were inoculated into 150 mL pre-cultures of Terrific Broth (TB) medium containing 100 μg/mL carbenicillin, 50 μg/mL kanamycin, and 50 μg/mL chloramphenicol for ~48 h (without ALA). The pre-cultures were transferred into 10 glass flasks, each containing 300 mL of TB medium supplemented with the above antibiotics to an initial OD600 (optical density) of 0.1. The expression of TgMQO was induced by the addition of 100 μM isopropyl β-D-thiogalactopyranoside (Sigma, Steinheim, Germany) and cultured at 30 °C on a rotary shaker (200 rpm; Bioshaker BR-43FL; Taitec, Saitama, Japan) until the OD600 reached 4.0 to 5.0 (48-72 h). All of the following processes were performed at 4 °C. The cells were harvested by centrifugation at 5000× *g* for 15 min, the pellet was resuspended in lysis buffer [50 mM MOPS (Dojindo, Kumamoto, Japan) pH 8.0 and 250 μM phenylmethylsulfonyl fluoride (PMSF, Wako)], and cells were lysed by a single passage through a French Press (Ohtake) at 180 MPa. Unbroken cells and debris were removed by centrifugation at 30,000× *g* for 30 min (R20A2 rotor, Hitachi, Tokyo, Japan), and the supernatant was ultracentrifuged at 200,000× *g* for 1 h (45TI rotor, Hitachi). The crude membrane pellet was homogenized in a buffer containing 50 mM MOPS pH 8.0, 5 mM imidazole (Wako), 150 mM KCl (Wako), and 200 μM flavin adenine dinucleotide (FAD, Sigma) and then stored in 50% (*v*/*v*) glycerol (Wako) at −30 °C until purification. The membrane fraction from FN102(DE3)TAO harboring empty pET151 was cultured and prepared in parallel for use as a control.

### 4.3. Purification of TgMQO from the Membrane Fraction

The membrane fraction at a concentration of 9.4 mg/mL was diluted (1:1 volume ratio) with dilution buffer (50 mM MOPS pH 8.0, 200 μM FAD) and mixed with solubilizing buffer [50 mM MOPS pH 8.0, 2% (*w*/*v*) n-octyl-β-D-glucopyranoside (OG, Dojindo), 10 mg/mL 1,2-diacyl-sn-glycero-3-phosphocholine (Sigma), 200 μM FAD] at a 1:1 volume ratio, followed by gentle rotation (Rotator RT-50, Taitec) for 30 min. The solubilized membrane was subjected to ultracentrifugation (S50A-2224 rotor, Hitachi CS150FNX) at 200,000× *g* for 1 h to remove the insoluble proteins. The supernatant was mixed with 1.5 mL cOmplete^TM^ His-Tag Purification Resin (Roche, Mannheim, Germany) pre-equilibrated with wash buffer A [50 mM MOPS pH 8.0, 0.1% (*w*/*v*) OG, 200 μM FAD, 1.5 mM imidazole] and kept rotating for 2 h. The mixture was loaded onto a Poly-Prep^®^ Chromatography column (20 mL, Bio-Rad, Hercules, CA, USA), and the unbound protein was collected as flow-through. The column was washed with 20 volumes (30 mL) of wash buffer A followed by 30 mL of wash buffer B [50 mM MOPS pH 8.0, 0.1% (*w*/*v*) OG, 20 mM imidazole, 200 μM FAD]. Finally, the recombinant TgMQO was eluted with 15 mL of elution buffer [50 mM MOPS pH 8.0, 0.1% (*w*/*v*) OG, 200 μM FAD, 200 mM imidazole]. A centrifugal filter device (Amicon Ultra-15, 30 kDA cutoff, Millipore, Tullagreen, Ireland) was used to concentrate the eluted fractions. The concentrated TgMQO was mixed with glycerol and FAD to final 50% (*v*/*v*) and 200 µM, respectively, and stored at −30 °C until use.

### 4.4. Protein Quantification and Electrophoresis

Protein concentrations were determined at 595 nm using a Bio-Rad Protein Assay kit with bovine serum albumin (Takara, Shiga, Japan) as the standard according to the manufacturer’s protocol.

Fractions from each purification step were subjected to discontinuous SDS-PAGE [93]. The stacking and separating gels were 4% and 12% (*w*/*v*) acrylamide, respectively. Samples were mixed with SDS-PAGE loading buffer [62.5 mM Tris-HCl pH 6.8, 2.5% (*v*/*v*) sodium dodecyl sulphate (SDS, Wako), 10% (*v*/*v*) glycerol, 0.002% (*v*/*v*) bromophenol blue (Wako), and 0.71 M β-mercaptoethanol (Wako)] to a final concentration of 1 mg/mL (except for wash 2 and concentrated sample) and heated for 10 min at 95°C. Protein preparations were loaded at 10 μg per well (except for wash 2 and concentrated sample) onto the gel and run alongside broad-ranged Precision Plus Protein^TM^ Standards (Bio-Rad). The electrophoresis was carried out at 25 mA for 90 min at room temperature, and the gel was washed three times for 5 min with purified water and stained with GelCode^TM^ Blue Safe Protein stain (Thermo Fisher Scientific, Waltham, MA, USA) according to the provider’s manual.

hrCNE was performed by adapting the reported protocol for DHODH [88]. Briefly, purified TgMQO was diluted to 0.3, 0.1, and 0.03 mg/mL in 50 mM MOPS pH 8.0, 0.05% (*v*/*v*) n-dodecyl β-D-maltoside (DDM, Sigma), 0.05% (*v*/*v*) sodium deoxycholate (DOC, Nacalai Tesque, Kyoto, Japan), 0.0001% (*w*/*v*) ponceau S (MP Biomedicals LLC, Illkirch, France), and 5% (*v*/*v*) glycerol (final concentrations). The samples (3, 1, and 0.3 μg/lane) were loaded onto a NativePAGE^TM^ 4–16% (*w*/*v*) Bis-Tris gradient gel (Invitrogen) along with NativeMark^TM^ (Invitrogen) as a protein standard in duplicate. The gel was run at 4 °C under a constant voltage of 150 V for approximately 90 min in NativePAGE^TM^ 20× running buffer (Novex, Invitrogen) according to the manufacturer’s manual. The cathode buffer was also supplemented with 0.05% (*v*/*v*) DDM and 0.05% (*v*/*v*) DOC. The gel was split into two sets for GelCode^TM^ Blue Safe Protein and MQO activity stainings. The in-gel MQO-activity staining was performed by washing three times with 5 mM MOPS pH 8.0 for 5 min each at room temperature. After washing, the gel was incubated with 1.5 mM nitro-blue tetrazolium chloride (NBT, Wako) dissolved in 5 mM MOPS pH 8.0 for 5 min at room temperature. Phenazine methyl sulfate (PMS, TCI) and malate (Wako) were added to a final concentration of 0.1 mg/mL and 10 mM, respectively, mixed quickly, kept static in the dark until the bands became visible, and subsequently washed with purified water.

### 4.5. Optimization of TgMQO Assay Conditions

The dose response range of partially purified TgMQO was assayed at 37 °C using a UV760 (Jasco-Japan, Tokyo, Japan) at different concentrations of purified TgMQO, ranging from 0.025 to 5 µg/mL, in 1 mL of the reaction mixture as previously described [23] in triplicate, with a minor change of KCN replaced with 50 nM AF.

Optimization of temperature for the TgMQO assay was performed spectrophotometrically using a UV760 (Jasco-Japan) equipped with a water bath circulator (Taitec). The TgMQO activity was assayed using a 1 mL black quartz cuvette with 50 mM HEPES pH 7.0, 120 μM DCIP, 50 nM AF, 20 μM dUQ, and 1.5 μg/mL purified TgMQO at varying temperatures in triplicate. The reaction was started by the addition of 10 mM malate, and the activity was calculated as described previously [23].

The optimum pH was determined by measuring TgMQO activity at different pHs using 50 mM of sodium phosphate (NaPi, pH 5.8–8.0), potassium phosphate (KPi, pH 5.8–8.0), HEPES-NaOH (6.8–8.4), Tris-HCl (Wako) (pH 6.9–9.0), MOPS-NaOH (Dojindo) (pH 6.5–9.0), or CHES-NaOH (Dojindo) (pH 8.6–10.0) buffers at 37 °C with a SpectraMax^®^ Paradigm^®^ Multi-Mode Microplate Reader (Molecular Devices, San Jose, CA, USA), as described previously [23].

### 4.6. Determination of Enzyme Kinetic Parameters and Reaction Mechanism

Kinetic parameters of different ubiquinones (UQ0, UQ1, UQ2, UQ4, UQ6, and dUQ) and malate, and the reaction mechanism of TgMQO were determined at 37 °C and pH 7.0 by the direct consumption of dUQ at 278 nm (ε_278_ = 15 mM^−1^·cm^−1^) over 5 min in triplicate. The concentration of TgMQO was fixed at 0.0375 µg/mL, and *K*_m_ and *V*_max_ were determined by varying concentrations of different ubiquinones from 1 to 100 µM at 10 mM malate. Similarly, the *K*_m_ and *V*_max_ of malate were determined at varying concentrations ranging between 0.5 and 100 mM using 20 µM of different ubiquinones (UQ1, UQ2, and dUQ) as electron acceptors. The data were analyzed and kinetic constants calculated using GraphPad Prism 7.0 software (San Diego, CA, USA).

For comparison with PfMQO, the reaction mechanism of TgMQO was analyzed using malate/dUQ as substrates following a previous protocol [23]. In short, TgMQO activity was assayed at varying dUQ concentrations (2, 4, 6, 8, 10, 12, 14, 16, 18, and 20 µM) under malate concentrations of 2, 10, and 20 mM, in single experiments, and the *K*_m_ of dUQ was determined at each malate concentration.

### 4.7. TgMQO Inhibition with Ferulenol

The concentration of ferulenol (AdipoGen, Liestal, Switzerland) inhibiting 50% of the TgMQO activity (IC_50_) was determined at 278 nm using a 1 mL black quartz cuvette. The reaction buffer contained 50 mM HEPES-NaOH pH 7.0, 50 nM AF, 20 µM dUQ, and 0.0375 µg/mL of TgMQO, and enzymatic activity was measured in the presence of different concentrations of ferulenol. The reaction was initiated by the addition of 10 mM malate. The IC_50_ and the *K*_i_^app^ were calculated as previously described [94].

The inhibition mechanism versus dUQ was determined using a reaction mix containing 50 mM HEPES pH 7.0, 50 nM AF, and 0.0375 µg/mL TgMQO. TgMQO activity was measured at different dUQ concentrations (2, 4, 6, 8, 10, 12, 14, 16, 18, 20, 22, and 24 µM) under 0, 0.5, 1, and 2 µM of ferulenol. The inhibition mechanism versus malate was determined using different malate concentrations (0.5, 1, 2, 4, 6, 8, 10, 12, 14, 16, 18, and 20 mM) under a fixed dUQ concentration of 20 µM. In both cases, the activity was measured at 278 nm in single experiments, and the reaction started with 10 mM of malate. The data were analyzed using GraphPad Prism 7.0 software.

## Figures and Tables

**Table 1:** Kinetic constants of purified TgMQO.

Substrates		*K*_m_ (μM)	*V*_max_ (μmol/min/mg)
Variable	Fixed		
Malate	Ubiquinone-1	370 ± 140	6.30 ± 0.5
Malate	Ubiquinone-2	637 ± 180	24.6 ± 0.8
Malate	Decylubiquinone	466 ± 291	9.55 ± 0.6
Ubiquinone-0	Malate	225 ± 77	12.7 ± 3.2
Ubiquinone-1	Malate	116 ± 16	44.2 ± 3.8
Ubiquinone-2 ^1^	Malate	13.2 ^1^	45.1 ^1^
Ubiquinone-4	Malate	7.7 ± 4.3	4.39 ± 0.7
Ubiquinone-6	Malate	8.0 ± 3.3	3.64 ± 0.4
Decylubiquinone	Malate	17 ± 4.7	12.1 ± 1.2

Values represent the average ± SD (*n* = 3). ^1^ UQ2 showed substrate inhibition over 20 μM. So, the *K*_m_ and *V*_max_ calculated here were below the substrate inhibition concentration.

## Data Availability

Not applicable.

## Supplementary Materials

The following are available online at https://www.mdpi.com/article/10.3390/ijms22157830/s1.

## References

[1] Demar M., Ajzenberg D., Maubon D., Djossou F., Panchoe D., Punwasi W., Valery N., Peneau C., Daigre J.-L., Aznar C. (2007). Fatal outbreak of human toxoplasmosis along the Maroni River: Epidemiological, clinical, and parasitological aspects. Clin. Infect. Dis..

[2] Dubey J.P. (2016). Toxoplasmosis of Animals and Humans.

[3] Galal L., Schares G., Stragier C., Vignoles P., Brouat C., Cuny T., Dubois C., Rohart T., Glodas C., Dardé M.L. (2019). Diversity of *Toxoplasma gondii* strains shaped by commensal communities of small mammals. Int. J. Parasitol..

[4] Hejlícek K., Literák I., Nezval J. (1997). Toxoplasmosis in wild mammals from the Czech Republic. J. Wildl. Dis..

[5] Flegr J., Prandota J., Sovičková M., Israili Z.H. (2014). Toxoplasmosis—A Global Threat. Correlation of latent toxoplasmosis with specific disease burden in a set of 88 countries. PLoS ONE.

[6] Dubremetz J.F., Lebrun M. (2012). Virulence factors of *Toxoplasma gondii*. Microbes Infect..

[7] Blume M., Seeber F. (2018). Metabolic interactions between *Toxoplasma gondii* and its host. F1000Research.

[8] Soête M., Camus D., Dubremetz J.F. (1994). Experimental induction of bradyzoite-specific antigen expression and cyst formation by the RH strain of *Toxoplasma gondii* in vitro. Exp. Parasitol..

[9] Weilhammer D.R., Iavarone A.T., Villegas E.N., Brooks G.A., Sinai A.P., Sha W.C. (2012). Host metabolism regulates growth and differentiation of *Toxoplasma gondii*. Int. J. Parasitol..

[10] Tomavo S., Boothroyd J.C. (1995). Interconnection between organellar functions, development and drug resistance in the protozoan parasite. Toxoplasma gondii. Int. J. Parasitol..

[11] Boothroyd J.C., Grigg M.E. (2002). Population biology of *Toxoplasma gondii* and its relevance to human infection: Do different strains cause different disease?. Curr. Opin. Microbiol..

[12] Ajzenberg D., Yera H., Marty P., Paris L., Dalle F., Menotti J., Aubert D., Franck J., Bessières M.H., Quinio D. (2009). Genotype of 88 *Toxoplasma gondii* isolates associated with toxoplasmosis in immunocompromised patients and correlation with clinical findings. J. Infect. Dis..

[13] Dardé M.L., Villena I., Pinon J.M., Beguinot I. (1998). Severe toxoplasmosis caused by a *Toxoplasma gondii* strain with a new isoenzyme type acquired in French Guyana. J. Clin. Microbiol..

[14] Hosseini S.A., Amouei A., Sharif M., Sarvi S., Galal L., Javidnia J., Pagheh A.S., Gholami S., Mizani A., Daryani A. (2018). Human toxoplasmosis: A systematic review for genetic diversity of *Toxoplasma gondii* in clinical samples. Epidemiol. Infect..

[15] Jones J.L., Kruszon-Moran D., Wilson M., McQuillan G., Navin T., McAuley J.B. (2001). *Toxoplasma gondii* Infection in the United States: Seroprevalence and Risk Factors. Am. J. Epidemiol..

[16] Dupont C.D., Christian D.A., Hunter C.A. (2012). Immune response and immunopathology during toxoplasmosis. Semin. Immunopathol..

[17] Ferguson D.J.P., Bowker C., Jeffery K.J.M., Chamberlain P., Squier W. (2013). Congenital toxoplasmosis: Continued parasite proliferation in the fetal brain despite maternal immunological control in other tissues. Clin. Infect. Dis..

[18] Montoya J.G., Liesenfeld O. (2004). Toxoplasmosis. Lancet.

[19] Konstantinovic N., Guegan H., Stäjner T., Belaz S., Robert-Gangneux F. (2019). Treatment of toxoplasmosis: Current options and future perspectives. Food Waterborne Parasitol..

[20] Sinai A.P., Watts E.A., Dhara A., Murphy R.D., Gentry M.S., Patwardhan A. (2016). Reexamining chronic *Toxoplasma gondii* infection: Surprising activity for a “dormant” parasite. Curr. Clin. Microbiol. Rep..

[21] Buxton D., Innes E.A. (1995). A commercial vaccine for ovine toxoplasmosis. Parasitology.

[22] Lyons R.E., McLeod R., Roberts C.W. (2002). Toxoplasma gondii tachyzoite-bradyzoite interconversion. Trends Parasitol..

[23] Hartuti E.D., Inaoka D.K., Komatsuya K., Miyazaki Y., Miller R.J., Xinying W., Sadikin M., Prabandari E.E., Waluyo D., Kuroda M. (2018). Biochemical studies of membrane bound *Plasmodium falciparum* mitochondrial L-malate:quinone oxidoreductase, a potential drug target. Biochim. Biophys. Acta Bioenerg..

[24] MacRae J.I., Sheiner L., Nahid A., Tonkin C., Striepen B., McConville M.J. (2012). Mitochondrial metabolism of glucose and glutamine is required for intracellular growth of *Toxoplasma gondii*. Cell Host Microbe.

[25] Polonais V., Soldati-Favre D. (2010). Versatility in the acquisition of energy and carbon sources by the Apicomplexa. Biol. Cell.

[26] Shukla A., Olszewski K.L., Llinás M., Rommereim L.M., Fox B.A., Bzik D.J., Xia D., Wastling J., Beiting D., Roos D.S. (2018). Glycolysis is important for optimal asexual growth and formation of mature tissue cysts by *Toxoplasma gondii*. Int. J. Parasitol..

[27] Fleige T., Pfaff N., Gross U., Bohne W. (2008). Localisation of gluconeogenesis and tricarboxylic acid (TCA)-cycle enzymes and first functional analysis of the TCA cycle in *Toxoplasma gondii*. Int. J. Parasitol..

[28] Hikosaka K., Komatsuya K., Suzuki S., Kita K. (2015). Mitochondria of malaria parasites as a drug target. An Overview of Tropical Diseases.

[29] Kawahara K., Mogi T., Tanaka T.Q., Hata M., Miyoshi H., Kita K. (2009). Mitochondrial dehydrogenases in the aerobic respiratory chain of the rodent malaria parasite *Plasmodium yoelii yoelii*. J. Biochem..

[30] Mather M.W., Henry K.W., Vaidya A.B. (2007). Mitochondrial drug targets in apicomplexan parasites. Curr. Drug. Targets.

[31] Mi-Ichi F., Miyadera H., Kobayashi T., Takamiya S., Waki S., Iwata S., Shibata S., Kita K. (2005). Parasite mitochondria as a target of chemotherapy: Inhibitory effect of licochalcone A on the *Plasmodium falciparum* respiratory chain. Ann. N. Y. Acad. Sci..

[32] Fueyo González F.J., Ebiloma G.U., Izquierdo García C., Bruggeman V., Sánchez Villamañán J.M., Donachie A., Balogun E.O., Inaoka D.K., Shiba T., Harada S. (2017). Conjugates of 2,4-dihydroxybenzoate and salicylhydroxamate and lipocations display potent antiparasite effects by efficiently targeting the *Trypanosoma brucei* and *Trypanosoma congolense* mitochondrion. J. Med. Chem..

[33] Shiba T., Kido Y., Sakamoto K., Inaoka D.K., Tsuge C., Tatsumi R., Takahashi G., Balogun E.O., Nara T., Aoki T. (2013). Structure of the trypanosome cyanide-insensitive alternative oxidase. Proc. Natl. Acad. Sci. USA.

[34] Yamasaki S., Shoji M., Kayanuma M., Sladek V., Inaoka D.K., Matsuo Y., Shiba T., Young L., Moore A.L., Kita K. (2021). Weak O_2_ binding and strong H_2_O_2_ binding at the non-heme diiron center of trypanosome alternative oxidase. Biochim. Biophys. Acta Bioenerg..

[35] Young L., Rosell-Hidalgo A., Inaoka D.K., Xu F., Albury M., May B., Kita K., Moore A.L. (2020). Kinetic and structural characterisation of the ubiquinol-binding site and oxygen reduction by the trypanosomal alternative oxidase. Biochim. Biophys. Acta Bioenerg..

[36] Seidi A., Muellner-Wong L.S., Rajendran E., Tjhin E.T., Dagley L.F., Aw V.Y.T., Faou P., Webb A.I., Tonkin C.J., van Dooren G.G. (2018). Elucidating the mitochondrial proteome of *Toxoplasma gondii* reveals the presence of a divergent cytochrome *c* oxidase. eLife.

[37] Hikosaka K., Kita K., Tanabe K. (2013). Diversity of mitochondrial genome structure in the phylum Apicomplexa. Mol. Biochem. Parasitol..

[38] Doggett J.S., Nilsen A., Forquer I., Wegmann K.W., Jones-Brando L., Yolken R.H., Bordón C., Charman S.A., Katneni K., Schultz T. (2012). Endochin-like quinolones are highly efficacious against acute and latent experimental toxoplasmosis. Proc. Natl. Acad. Sci. USA.

[39] Phillips M.A., Gujjar R., Malmquist N.A., White J., El Mazouni F., Baldwin J., Rathod P.K. (2008). Triazolopyrimidine-based dihydroorotate dehydrogenase inhibitors with potent and selective activity against the malaria parasite *Plasmodium falciparum*. J. Med. Chem..

[40] Seeber F., Limenitakis J., Soldati-Favre D. (2008). Apicomplexan mitochondrial metabolism: A story of gains, losses and retentions. Trends Parasitol..

[41] Lin S.S., Gross U., Bohne W. (2011). Two internal type II NADH dehydrogenases of *Toxoplasma gondii* are both required for optimal tachyzoite growth. Mol. Microbiol..

[42] Denton H., Roberts C.W., Alexander J., Thong K.W., Coombs G.H. (1996). Enzymes of energy metabolism in the bradyzoites and tachyzoites of *Toxoplasma gondii*. FEMS Microbiol. Lett..

[43] Jacot D., Waller R.F., Soldati-Favre D., MacPherson D.A., MacRae J.I. (2016). Apicomplexan energy metabolism: Carbon source promiscuity and the quiescence hyperbole. Trends Parasitol..

[44] Lin S.S., Gross U., Bohne W. (2009). Type II NADH dehydrogenase inhibitor 1-hydroxy-2-dodecyl-4(1H)quinolone leads to collapse of mitochondrial inner-membrane potential and ATP depletion in *Toxoplasma gondii*. Eukaryot. Cell.

[45] Bisanz C., Bastien O., Grando D., Jouhet J., Maréchal E., Cesbron-Delauw M.F. (2006). *Toxoplasma gondii* acyl-lipid metabolism: De novo synthesis from apicoplast-generated fatty acids versus scavenging of host cell precursors. Biochem. J..

[46] Shen W., Wei Y., Dauk M., Tan Y., Taylor D.C., Selvaraj G., Zou J. (2006). Involvement of a glycerol-3-phosphate dehydrogenase in modulating the NADH/NAD+ ratio provides evidence of a mitochondrial glycerol-3-phosphate shuttle in *Arabidopsis*. Plant Cell.

[47] Hortua Triana M.A., Cajiao Herrera D., Zimmermann B.H., Fox B.A., Bzik D.J. (2016). Pyrimidine pathway-dependent and -independent functions of the *Toxoplasma gondii* mitochondrial dihydroorotate dehydrogenase. Infect. Immun..

[48] Alday P.H., Doggett J.S. (2017). Drugs in development for toxoplasmosis: Advances, challenges, and current status. Drug Des. Devel. Ther..

[49] Hayward J.A., Rajendran E., Zwahlen S.M., Faou P., van Dooren G.G. (2021). Divergent features of the coenzyme Q:cytochrome c oxidoreductase complex in *Toxoplasma gondii* parasites. PLoS Pathog..

[50] Iltzsch M.H. (1993). Pyrimidine salvage pathways in *Toxoplasma gondii*. J. Eukaryot. Microbiol..

[51] Fox B.A., Bzik D.J. (2002). De novo pyrimidine biosynthesis is required for virulence of *Toxoplasma gondii*. Nature.

[52] Kather B., Stingl K., van der Rest M.E., Altendorf K., Molenaar D. (2000). Another unusual type of citric acid cycle enzyme in *Helicobacter pylori*: The malate:quinone oxidoreductase. J. Bacteriol..

[53] Bulusu V., Jayaraman V., Balaram H. (2011). Metabolic fate of fumarate, a side product of the purine salvage pathway in the intraerythrocytic stages of *Plasmodium falciparum*. J. Biol. Chem..

[54] Ke H., Lewis I.A., Morrisey J.M., McLean K.J., Ganesan S.M., Painter H.J., Mather M.W., Jacobs-Lorena M., Llinas M., Vaidya A.B. (2015). Genetic investigation of tricarboxylic acid metabolism during the *Plasmodium falciparum* life cycle. Cell Rep..

[55] Ke H., Morrisey J.M., Ganesan S.M., Painter H.J., Mather M.W., Vaidya A.B. (2011). Variation among *Plasmodium falciparum* strains in their reliance on mitochondrial electron transport chain function. Eukaryot. Cell.

[56] Painter H.J., Morrisey J.M., Mather M.W., Vaidya A.B. (2007). Specific role of mitochondrial electron transport in blood-stage *Plasmodium falciparum*. Nature.

[57] Mogi T., Murase Y., Mori M., Shiomi K., Omura S., Paranagama M.P., Kita K. (2009). Polymyxin B identified as an inhibitor of alternative NADH dehydrogenase and malate: Quinone oxidoreductase from the Gram-positive bacterium *Mycobacterium smegmatis*. J. Biochem..

[58] Molenaar D., van der Rest M.E., Petrović S. (1998). Biochemical and genetic characterization of the membrane-associated malate dehydrogenase (acceptor) from *Corynebacterium glutamicum*. Eur. J. Biochem..

[59] Molenaar D., van der Rest M.E., Drysch A., Yücel R. (2000). Functions of the membrane-associated and cytoplasmic malate dehydrogenases in the citric acid cycle of *Corynebacterium glutamicum*. J. Bacteriol..

[60] van der Rest M.E., Frank C., Molenaar D. (2000). Functions of the membrane-associated and cytoplasmic malate dehydrogenases in the citric acid cycle of *Escherichia coli*. J. Bacteriol..

[61] Niikura M., Komatsuya K., Inoue S.-I., Matsuda R., Asahi H., Inaoka D.K., Kita K., Kobayashi F. (2017). Suppression of experimental cerebral malaria by disruption of malate:quinone oxidoreductase. Malar. J..

[62] Wang X., Miyazaki Y., Inaoka D.K., Hartuti E.D., Watanabe Y.-I., Shiba T., Harada S., Saimoto H., Burrows J.N., Benito F.J.G. (2019). Identification of *Plasmodium falciparum* mitochondrial malate: Quinone oxidoreductase inhibitors from the pathogen Box. Genes.

[63] Araki Y., Awakawa T., Matsuzaki M., Cho R., Matsuda Y., Hoshino S., Shinohara Y., Yamamoto M., Kido Y., Inaoka D.K. (2019). Complete biosynthetic pathways of ascofuranone and ascochlorin in *Acremonium egyptiacum*. Proc Natl. Acad. Sci. USA.

[64] Hijikawa Y., Matsuzaki M., Suzuki S., Inaoka D.K., Tatsumi R., Kido Y., Kita K. (2017). Re-identification of the ascofuranone-producing fungus *Ascochyta viciae* as *Acremonium sclerotigenum*. J. Antibiot..

[65] Borisov V.B., Gennis R.B., Hemp J., Verkhovsky M.I. (2011). The cytochrome *bd* respiratory oxygen reductases. Biochim. Biophys. Acta Bioenerg..

[66] Forte E., Borisov V.B., Falabella M., Colaço H.G., Tinajero-Trejo M., Poole R.K., Vicente J.B., Sarti P., Giuffrè A. (2016). The terminal oxidase cytochrome *bd* promotes sulfide-resistant bacterial respiration and growth. Sci. Rep..

[67] Kita K., Konishi K., Anraku Y. (1984). Terminal oxidases of *Escherichia coli* aerobic respiratory chain. II. Purification and properties of cytochrome *b_558_-d* complex from cells grown with limited oxygen and evidence of branched electron-carrying systems. J. Biol. Chem..

[68] Nihei C., Fukai Y., Kawai K., Osanai A., Yabu Y., Suzuki T., Ohta N., Minagawa N., Nagai K., Kita K. (2003). Purification of active recombinant trypanosome alternative oxidase. FEBS Lett..

[69] Ohshima T., Tanaka S. (1993). Dye-linked L-malate dehydrogenase from thermophilic *Bacillus* species DSM 465. Purification and characterization. Eur. J. Biochem..

[70] Shinagawa E., Fujishima T., Moonmangmee D., Theeragool G., Toyama H., Matsushita K., Adachi O. (2002). Purification and characterization of membrane-bound Malate dehydrogenase from *Acetobacter* sp. SKU 14. Biosci. Biotechnol. Biochem..

[71] Oh Y.-R., Jang Y.-A., Hong S.H., Eom G.T. (2020). Purification and characterization of a malate:quinone oxidoreductase from P*seudomonas taetrolens* capable of producing valuable lactobionic acid. J. Agric. Food Chem..

[72] Kabashima Y., Sone N., Kusumoto T., Sakamoto J. (2013). Purification and characterization of malate:quinone oxidoreductase from thermophilic *Bacillus* sp. PS3. J. Bioenerg. Biomembr..

[73] Chrétien D., Bénit P., Ha H.-H., Keipert S., El-Khoury R., Chang Y.-T., Jastroch M., Jacobs H.T., Rustin P., Rak M. (2018). Mitochondria are physiologically maintained at close to 50 °C. PLoS Biol..

[74] Macherel D., Haraux F., Guillou H., Bourgeois O. (2021). The conundrum of hot mitochondria. Biochim. Biophys. Acta Bioenerg..

[75] Lindsay D.S., Mitschler R.R., Toivio-Kinnucan M.A., Upton S.J., Dubey J.P., Blagburn B.L. (1993). Association of host cell mitochondria with developing *Toxoplasma gondii* tissue cysts. Am. J. Vet. Res..

[76] Nelson M.M., Jones A.R., Carmen J.C., Sinai A.P., Burchmore R., Wastling J.M. (2008). Modulation of the host cell proteome by the intracellular apicomplexan parasite *Toxoplasma gondii*. Infect. Immun..

[77] Pernas L., Adomako-Ankomah Y., Shastri A.J., Ewald S.E., Treeck M., Boyle J.P., Boothroyd J.C. (2014). Toxoplasma effector MAF1 mediates recruitment of host mitochondria and impacts the host response. PLoS Biol..

[78] Sinai A.P., Webster P., Joiner K.A. (1997). Association of host cell endoplasmic reticulum and mitochondria with the *Toxoplasma gondii* parasitophorous vacuole membrane: A high affinity interaction. J. Cell Sci..

[79] Lahouel M., Zini R., Zellagui A., Rhouati S., Carrupt P.-A., Morin D. (2007). Ferulenol specifically inhibits succinate ubiquinone reductase at the level of the ubiquinone cycle. Biochem. Biophys. Res. Commun..

[80] Appendino G., Mercalli E., Fuzzati N., Arnoldi L., Stavri M., Gibbons S., Ballero M., Maxia A. (2004). Antimycobacterial coumarins from the sardinian giant fennel (*Ferula communis*). J. Nat. Prod..

[81] Bell R.G., Sadowski J.A., Matschiner J.T. (1972). Mechanism of action of warfarin. Warfarin and metabolism of vitamin K_1_. Biochemistry.

[82] Fasco M.J., Principe L.M., Walsh W.A., Friedman P.A. (1983). Warfarin inhibition of vitamin K 2,3-epoxide reductase in rat liver microsomes. Biochemistry.

[83] Sadler J.E. (2004). K is for koagulation. Nature.

[84] Bocca C., Gabriel L., Bozzo F., Miglietta A. (2002). Microtubule-interacting activity and cytotoxicity of the prenylated coumarin ferulenol. Planta Med..

[85] Gebauer M. (2007). Synthesis and structure–activity relationships of novel warfarin derivatives. Bioorganic Med. Chem..

[86] Li W., Schulman S., Dutton R.J., Boyd D., Beckwith J., Rapoport T.A. (2010). Structure of a bacterial homologue of vitamin K epoxide reductase. Nature.

[87] Huang L.-s., Luemmen P., Berry E. (2021). Crystallographic investigation of the ubiquinone binding site of respiratory Complex II and its inhibitors. Biochim. Biophys. Acta Proteins Proteom..

[88] Sato D., Hartuti E.D., Inaoka D.K., Sakura T., Amalia E., Nagahama M., Yoshioka Y., Tsuji N., Nozaki T., Kita K. (2020). Structural and biochemical features of *Eimeria tenella* dihydroorotate dehydrogenase, a potential drug target. Genes.

[89] Balogun E.O., Inaoka D.K., Shiba T., Tsuge C., May B., Sato T., Kido Y., Nara T., Aoki T., Honma T. (2019). Discovery of trypanocidal coumarins with dual inhibition of both the glycerol kinase and alternative oxidase of *Trypanosoma brucei brucei*. FASEB J..

[90] Shiba T., Inaoka D.K., Takahashi G., Tsuge C., Kido Y., Young L., Ueda S., Balogun E.O., Nara T., Honma T. (2019). Insights into the ubiquinol/dioxygen binding and proton relay pathways of the alternative oxidase. Biochim Biophys. Acta Bioenerg..

[91] Holdgate G.A., Meek T.D., Grimley R.L. (2018). Mechanistic enzymology in drug discovery: A fresh perspective. Nat. Rev. Drug Dis..

[92] Ebiloma G.U., Ayuga T.D., Balogun E.O., Gil L.A., Donachie A., Kaiser M., Herraiz T., Inaoka D.K., Shiba T., Harada S. (2018). Inhibition of trypanosome alternative oxidase without its N-terminal mitochondrial targeting signal (ΔMTS-TAO) by cationic and non-cationic 4-hydroxybenzoate and 4-alkoxybenzaldehyde derivatives active against *T. brucei* and *T. Congolense*. Eur. J. Med. Chem..

[93] Laemmli U.K. (1970). Cleavage of structural proteins during the assembly of the head of bacteriophage T4. Nature.

[94] Inaoka D.K., Iida M., Tabuchi T., Honma T., Lee N., Hashimoto S., Matsuoka S., Kuranaga T., Sato K., Shiba T. (2016). The open form inducer approach for structure-based drug design. PLoS ONE.

