# Supplementary material for: Biochemical Studies of Mitochondrial Malate: Quinone Oxidoreductase from Toxoplasma gondii
Source: Int J Mol Sci. 2021 Jul 22;22(15):7830. doi: 10.3390/ijms22157830 (PMC8345934; doi:10.3390/ijms22157830)
Supplement: Supplementary file 1 [file ijms-22-07830-s001.zip › ijms-1289193-supplementary.pdf]

International Journal of
Molecular Sciences

MDPI

Supplementary table and figures

Table S1. Purification table of TgMQO.

Fraction	Total Protein (mg)	Total Activity [1] (μmol/min)	Yield * (%)	Specific Activity [2] (μmol/min/mg)	Purification # (x-fold)
Lysate	814	1702	100	2.1 ± 0.4	1
Clarified lysate	779	568	33	0.7 ± 0.2	0.33
Membrane	177	511	30	2.9 ± 0.5	1.4
Flow-through	98	333	20	3.4 ± 0.7	1.6
Elution	3.7	119	7.0	22 ± 0.7	10.5
Control membrane [3]	8.1	0.16	NA [4]	0.02 ± 0.005	NA

Yield * and Purification fold # are relative to the total activity and specific activity, respectively. [1] Activity was measured by DCIP reduction at 600 nm, and the data shown here are the averages of triplicate assays.[2] Specific activity measured in presence of 50 mM MOPS buffer pH 7.0 at 37 °C; the value represents the average ± SD (*n* = 3). [3] FN102(DE3)TAO/pET151 empty vector membrane fraction. [4] NA: Not applicable.

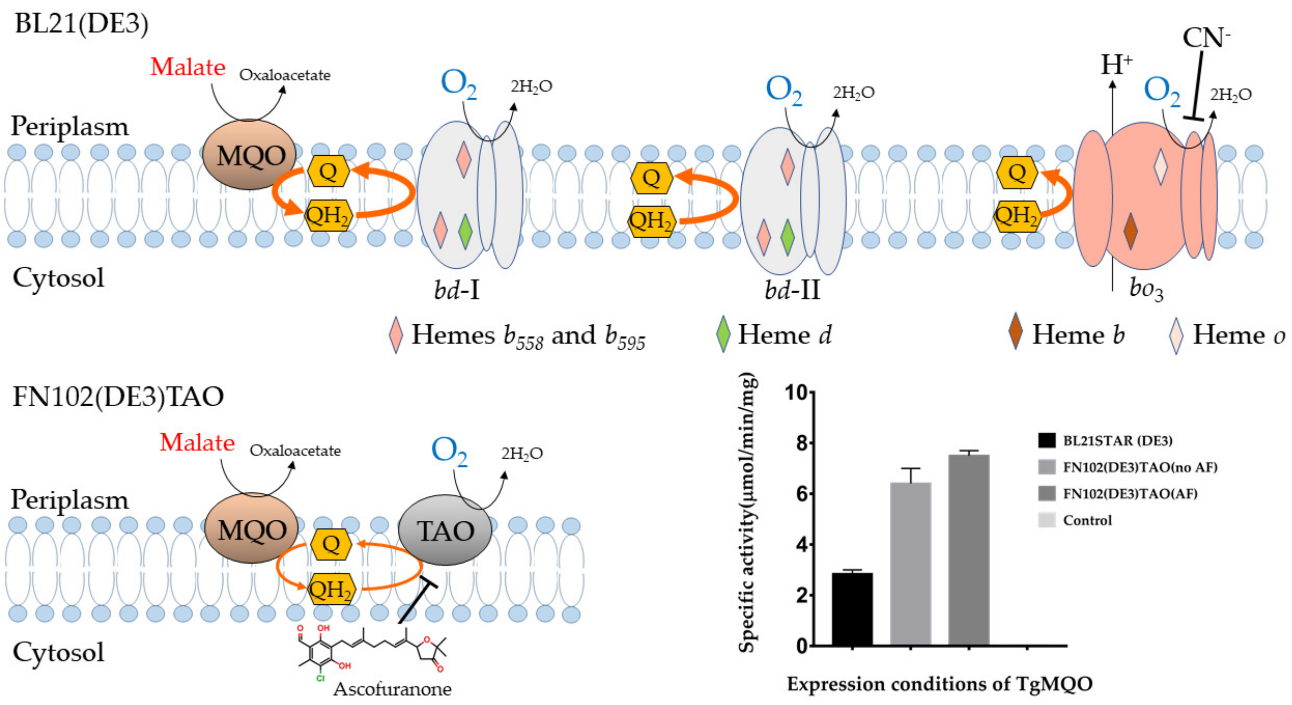

Figure S1. The electron transport chain (ETC) terminal oxidases of BL21(DE3) (top) and FN102(DE3)TAO (bottom left). The BL21(DE3) strain expresses heme-dependent terminal quinol oxidases (*bd*-I, *bd*-II, and *bo*3). Unlike *bo*3, *bd*-I and *bd*-II are cyanide-insensitive, which makes this expression system inappropriate to accurately measure the ubiquinone (Q) reductase activity of recombinant ETC dehydrogenases. This shortfall can be overcome with heme-deficient FN102(DE3)TAO (bottom left), in which the endogenous terminal oxidases are absent but complemented by the expression of trypanosome alternative oxidase (TAO). Ascofuranone (AF) is then used as a potent and specific inhibitor of TAO, avoiding the reoxidation of ubiquinol (QH_2). The specific activities of recombinant TgMQO expressed in the membrane fractions of different *E. coli* strains were also measured in presence of 50 mM HEPES buffer pH 7.0 (bottom right).

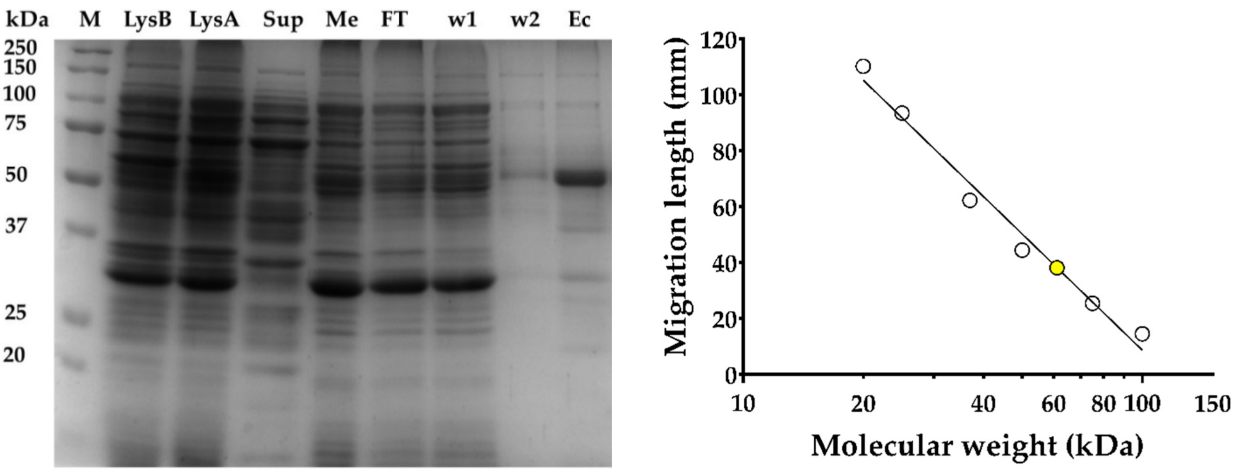

(**a**) (**b**)

Figure S2. SDS-PAGE analysis of the steps of TgMQO purification. (**a**) Fractions collected from each purification step of TgMQO. M = protein marker, 5 µl; LyB = lysate, 10 µg; LyA = clarified lysate, 10 µg; Sup = 200,000× *g* supernatant, 10 µg; Me = membrane fraction, 10 µg; FT = flow-through, 10 µg; w1 = wash 1, 10 µg; w2 = wash 2, 0.75 µg; Ec = elute concentrated, 4.2 µg. (**b**) Logarithmic plot (migration length vs. molecular weight) of SDS-PAGE. The yellow dot shows the estimated molecular weight of purified TgMQO (61 kDa).

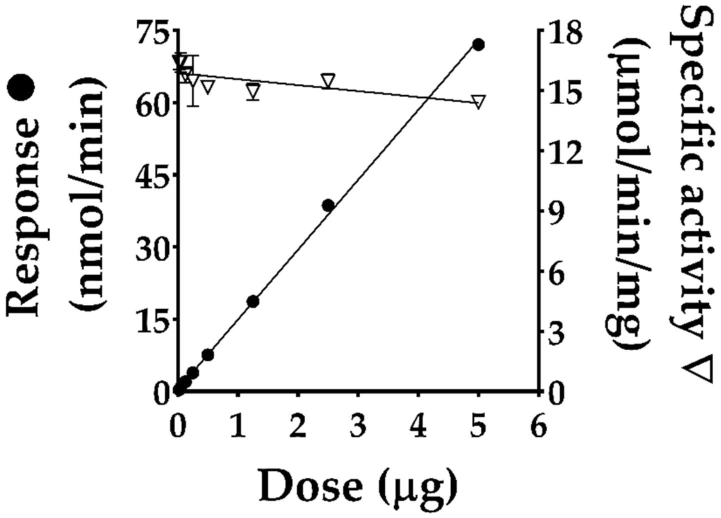

Figure S3. Determination of purified TgMQO dose response. MQO activity was assayed under varying concentrations of purified TgMQO, ranging from 0.025 µg/ml to 5 µg/ml in a 1-ml reaction mixture containing 50 mM HEPES pH 7.0, 120 µM DCIP, 20 µM dUQ, and 50 nM AF at 37°C. After recording the background, the reaction was initiated by adding 10 mM malate. The activity (nmol/min) and specific activity (µmol/min/mg) are shown on the left and right axes, respectively. Error bars are ± SD (n = 3). Purified TgMQO showed a linear dose response (R^2 = 0.9997) over the range tested.

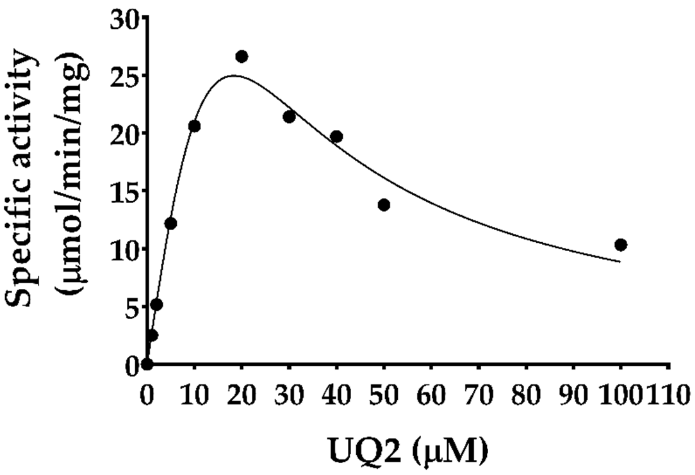

Figure S4. Inhibition of TgMQO enzymatic activity at high concentrations of UQ2. UQ2 concentrations over 20 µM show steep inhibition of TgMQO activity at a fixed concentration of 10 mM malate.

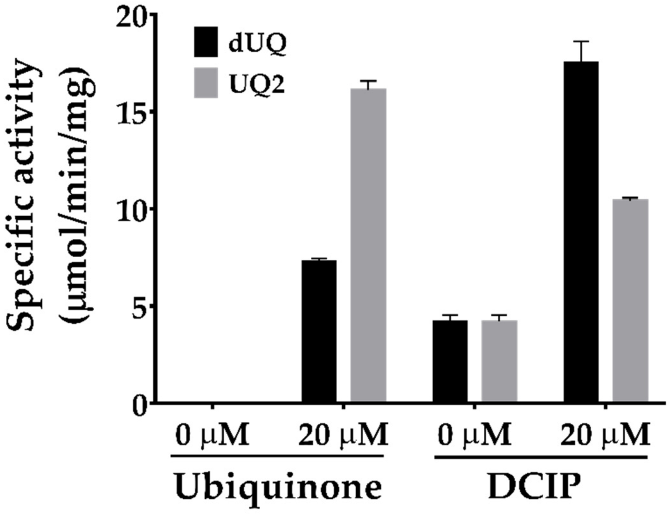

Figure S5. TgMQO activity assayed by ubiquinone (278 nm) or DCIP (600 nm) detection. No ubiquinone reduction could be detected without dUQ and UQ2. TgMQO showed higher activity with UQ2 than with dUQ as an electron acceptor. In the absence of the quinones, DCIP could be directly reduced by TgMQO. In the presence of quinones, DCIP-linked activity was higher for dUQ than UQ2, probably caused by the higher solubility of dUQ.
